# Relationship of chronic endometritis with chronic deciduitis in cases of miscarriage

**DOI:** 10.1186/s12905-020-00982-y

**Published:** 2020-06-01

**Authors:** Shoji Kaku, Takuro Kubo, Fuminori Kimura, Akiko Nakamura, Jun Kitazawa, Aina Morimune, Akimasa Takahashi, Akie Takebayashi, Akiko Takashima, Ryoji Kushima, Takashi Murakami

**Affiliations:** 1 Department of Obstetrics and Gynecology, Shiga University of Medical Science, Seta Tsukinowa-cho, Otsu, Shiga 520-2192 Japan; 2 Department of Obstetrics and Gynecology, National Hospital Organization Shiga Hospital, 255 Gochi-cho, Higashioumi, Shiga 527-8505 Japan; 3 Department of Clinical Laboratory Medicine and Division of Diagnostic Pathology, Shiga University of Medical Science, Seta Tsukinowa-cho, Otsu, Shiga 520-2192 Japan

**Keywords:** Chronic endometritis, Chronic deciduitis, Miscarriage

## Abstract

**Background:**

The presence of chronic deciduitis (CD) was determined in patients diagnosed with or without chronic endometritis (CE) before pregnancy.

**Objective:**

To study the effect of CE on decidua in cases of miscarriage.

**Methods:**

Decidual tissue was obtained from the patients who miscarried at the first pregnancy within a year after the diagnosis of the presence or absence of CE. The number and distribution pattern of plasma cells stained with CD138 in decidual tissue in 10 high-power fields (HPFs) was examined. The prevalence of CD diagnosed with four different grade; grade 0, no plasma cell in 10 HPFs, thus Non-CD;grade 1, rare single plasma cells; grade 2, rare clusters or more than 5 single cells total; and grade 3, many plasma cells with more than 5 clusters, were examined and compared between Non-CE and CE.

**Results:**

The incidence rate of CD of grade2 + 3 was significantly higher in CE than Non-CE (53.8%; 7/13 vs. 0%; 0/13, *P* < 0.01). Presence of clusters or a number of plasma cells in 10 HPFs of decidua showed a sensitivity of 53.8%, a specificity of 100%, a positive predictive value of 100%, and a negative predictive value of 68.4% for the diagnosis of CE.

**Conclusion:**

Presence of clusters of plasma cells or five or more of plasma cells in decidua was found in more than half of CE, but not found in Non-CE. When CD with cluster or five or more of plasma cells is confirmed histologically in miscarriage decidual tissue, the presence of CE before the pregnancy should be suspected.

## Background

Chronic endometritis (CE) is a slight inflammation of the endometrium that is histologically diagnosed by the presence of plasma cells in the stroma of the endometrium [1–5]. Several recent reports have shown that CE is associated with infertility, implantation failure, and habitual abortion [6–11]. In addition, it has been reported that the ongoing pregnancy rate is restored when CE is cured with antibiotic treatment, suggesting that the cause of CE is microbial infection [9, 12–15]. The features of the endometrium in CE patients include an increase in the cytotoxic NK cell ratio [16], dysfunction of decidualization [17], and an abnormal pattern of endometrial peristalsis [18], leading to infertility and implantation disorder. These physiological features before pregnancy may continue even after pregnancy and may be present in the decidua. However, so far, there has been no reports of how the endometrium of CE patients changes during pregnancy. The present study focused on chronic deciduitis (CD) for the purpose of histologically examining the effects of CE on the decidua. CD is defined as a type of long-term and slight inflammation of the decidua found during pregnancy [19–23]. Chronic microbial infection and immune mechanisms have been implicated as the etiology of CD [19, 24, 25]. The diagnosis of CD is similar to that of CE, depending histologically on the presence of plasma cells in the decidua [19, 20].

In the present study, the effect of CE on the decidua was determined by examining for the presence of plasma cells and the incidence of CD, using the decidual tissue of patients who became pregnant but miscarried following diagnosis with or without CE.

## Methods

This research was approved by the Ethics Committee of Shiga Medical University. Informed consent was obtained from the patients. The period of ovulation was identified by a urine ovulation test and vaginal ultrasonography, and endometrial tissue around the center of the anterior endometrium was collected with 4.5 J.A.M.W Type Uterine Curettes 5–9 days after ovulation from September 2013 to May 2018. Immunostaining with CD138 for endometrial tissue was performed according to previous reports [17, 26]. One of the gynecologists familiar with pathology judged the presence or absence of CD138-positive plasma cells and diagnosed CE when one or more CD138-positive plasma cells were found in 10 HPFs (HPF = field magnified 400 times with a microscope). Non-CE (control group) was defined when plasma cells were not found in 10 visual fields. Patient information was obtained from the medical charts. Patients who then underwent dilatation and curettage due to miscarriage of the first pregnancy within a year after the diagnosis of the presence or absence of CE were included in the present study. Patients who became pregnant after antibiotic treatment following the diagnosis of CE were excluded. The specimens of miscarriage tissue were immunostained with CD138 in the same manner as endometrial tissue, and the number of plasma cells in 10 HPFs of decidual tissue was counted. When one or more plasma cells were recognized in decidua, CD was diagnosed. CD was divided into four grades according to the distribution pattern and number of plasma cells in 10 HPFs: Grade 0, no plasma cell in 10 HPFs, thus Non-CD; Grade 1, 1 to 5 plasma cells in 10 HPFs; Grade 2, rare clusters or 5 to 20 plasma cells in 10 HPFs; and Grade 3, 20 or more plasma cells with more than 5 clusters in 10 HPFs. The number of plasma cells in 10 HPFs of decidual tissue and the prevalence of CD in patients with or without CE were examined. In addition, the percentage with CE was examined in Non-CD and in CD cases.

We calculated the number of patients required for enrollment using software provided by the Department of Biostatistics, Vanderbilt University (http://biostat.mc.vanderbilt.edu/wiki/Main/PowerSampleSize). Independent, case-control, two proportion, and Fisher’s exact test were selected to measure the sample size in the section of Dichotomous. We selected 0.05 for α (the probability that we will falsely reject the null hypothesis), 0.8 for power (β) (the probability of always rejecting the null hypothesis if the null hypothesis is false in the statistical hypothesis test), 0 for P0 (the probability of the outcome for a control patient in prospective studies), and 0.538 for P1 (the probability of the outcome in an experimental subject in prospective studies). When we chose 1 for m (the ratio of control to experimental subjects for independent prospective studies), the calculation resulted in sample sizes of 12 cases for the control group and 12 cases for the affected group. The numbers of the present study were thus adequate.

Statistical analysis was performed using Graph Pad Prism 5 (GraphPad Software Inc., La Jolla, CA). Each dataset was analyzed for a normal distribution using the Kolmogorov-Smirnov test, and Student’s *t*-test or the non-parametric Mann-Whitney U test was used depending on the distribution pattern. The significance of differences in the pregnancy rate, live birth rate, and miscarriage rate between the Non-CE group and the CE group was examined using Fisher’s analysis. A significant difference was considered present when the *P* value was less than 0.05.

## Results

**Fig. 1:**
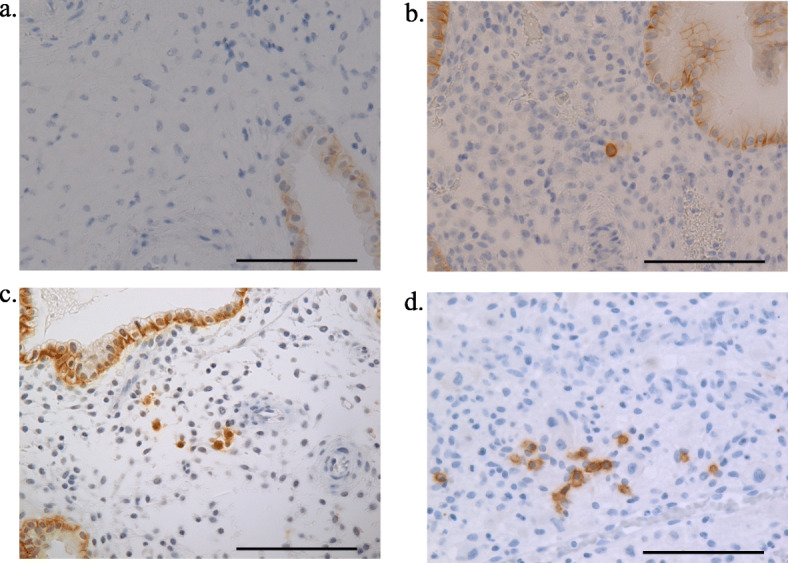
Immunohistochemistry of CD138 for decidual tissue. **a** No cells stained with CD138 are found in Non-CD. **b**. Grade 1 CD, one to 5 plasma cells found in 10 HPFs. **c**. Grade 2 CD, rare clusters or 5 to 20 plasma cells in 10 HPFs. **d**. Grade 3 CD, 20 or more plasma cells with more than 5 clusters in 10 HPFs. Bar = 100 μm

**Fig. 2:**
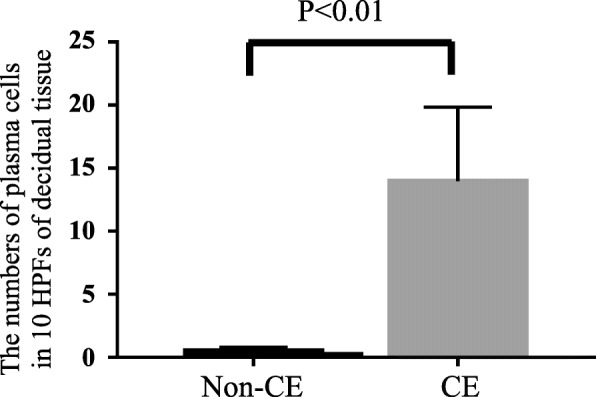
The number of plasma cells in the decidual tissue in Non-CE and CE patients. The numbers of plasma cells (mean ± standard error) in 10 HPFs of decidual tissue is 0.52 ± 0.17 and 14.0 ± 5.88 (P < 0.01) in Non-CE and CE patients, respectively.

Thirteen patients diagnosed with Non-CE became pregnant, but miscarried (Control; Non-CE group), and 13 patients who were diagnosed with CE and subsequently became pregnant, but miscarried (CE group) were enrolled. There were no differences in age, gravidity, parity, and gestational weeks at the time of dilatation and curettage between the Non-CE and CE groups (Table 1). The numbers of plasma cells (mean ± standard error of the mean) in 10 HPFs of decidual tissue were 0.54 ± 0.24 and 14.0 ± 5.88 (*P* < 0.01) in the Non-CE and CE groups, respectively (Fig. 1a, b, c, d, Fig. 2). Grade 1 CD was found in the Non-CE group, and Grade 1, 2, and 3 CD were found in the CE group (Table 1). The ratios of Grade 1 CD were 30.8% (4/13) and 15.4% (2/13) (*P* = 0.64) in the Non-CE group and CE group, respectively (Table 1). Similarly, the ratios of Grade 2 CD were 0% (0/13) and 30.8% (4/13) (*P* = 0.48), respectively, and the proportions of Grade 3 CD were 0% (0/13) and 23.1% (0/13) (*P* = 0.22), respectively (Table 1). The ratios of CD when defined as Grade 1 + Grade 2 + Grade 3 CD were 30.8% (4/13) and 69.2% (9/13) (*P* = 0.12), and the rates of Grade 2 + Grade 3 CD were 0% (0/13) and 53.8% (7/13) (*P* < 0.01) in the Non-CE group and CE group, respectively (Table 1). Of these, only the rates of Grade 2 + Grade 3 CD were significantly different between the two groups.

**Table 1:** Patients’ characteristics and the prevalence of CD by grade in the Non-CE and CE groups

		Non-CE	CE	***P*** value
		***N*** = 13	***N*** = 13	
Age, y, mean ± SEM		37.31 ± 1.11	37.31 ± 1.12	.99
Gravidity, mean ± SEM		2.15 ± 0.32	1.46 ± 0.14	.15
Parity, mean ± SEM		0.38 ± 0.14	0.23 ± 0.12	.67
Gestational weeks at the time of miscarriage, mean ± SEM		8w6.25d ± 1.74d	9w0.46d ± 0.84d	.53
CD	Grade 0 (%) Grade 1 (%)	9 (69.2) 4 (30.8)	4 (30.8) 2 (15.4)	.12 .64
	Grade 2 (%)	0 (0)	4 (30.8)	.48
	Grade 3 (%)	0 (0)	3 (23.1)	.22
	Grade 1 + 2 + 3 (%)	4 (30.8)	9 (69.2)	.12
	Grade 2 + 3 (%)	0 (0)	7 (53.8)	<.01
Cause of infertility	Male factor	3	3	
	Tubal factor	3	3	
	Endometriosis	2	2	
	Ovarian factor	3	0	
	Antisperm antibody	0	0	
	Fertilization failure	0	0	
	Unknown	2	5	

*CD* Chronic deciduitis, *CE* Chronic Endometritis, *SEM* Standard error of mean

The prevalence of CE before pregnancy was examined according to the presence or absence of CD. When CD was defined as the total of Grade 1 + Grade 2 + Grade 3, the ratios of CE before pregnancy were 30.8% (4/13) and 69.2% (9/13) (*P* = 0.12) in the Non-CD group and CD group. Similarly, when it was defined as Grade 2 + Grade 3, the ratios were 31.6% (6/19) and 100% (7/7 3) (*P* < 0.01), respectively. The ratios were 43.5% (10/23) and 100% (3/3) (*P* = 0.22), respectively, when it was only Grade 3.

With respect to the diagnosis of CE, Grade 2 + Grade 3 CD showed a sensitivity of 53.8%, a specificity of 100%, a positive predictive value of 100%, and a negative predictive value of 68.4%.

## Discussion

According to the results of the present study, CD with the presence of a cluster or a number of plasma cells was frequently found when a CE patient became pregnant and miscarried. This suggested that inflammation in the endometrium remains in the decidua of some cases of miscarriage.

CD is pathologically diagnosed with the presence of plasma cells within the decidua (decidual endometrium). Although studies of CD in preterm and/or term pregnancy have been reported [20, 23], there have been no reports of CD in early-stage pregnancy. Therefore, there are no diagnostic criteria for CD in early pregnancy. Gilmore et al. pointed out that the histologic diagnostic criteria for CE differ from the literature and used a semiquantitative scoring system to avoid these problems [27]. They divided the status into 4 grades: grade 0, no plasma cells seen; grade 1, rare single plasma cells; grade 2, rare clusters or more than 5 single cells total; and grade 3, many plasma cells with more than 5 clusters. In the present study, CD for the specimens of miscarriage cases was classified based on the report by Gilmore et al. That is, Non CD was defined as no plasma cells seen, and CD was divided into 3 grades: grade 1, 1 to 5 plasma cells in 10 HPFs; grade 2, rare clusters or 5 to 20 plasma cells in 10 HPFs; and grade 3, 20 or more plasma cells with more than 5 clusters in 10 HPFs. It was found that there were no significant differences in the incidence of CD of Grade 1, Grade 2, Grade 3, or Grade 1 + Grade 2 + Grade 3 between the Non-CE and CE groups. However, there was a significant difference in the incidence of Grade 2 + Grade 3 CD. In addition, although the number of cases was small in this study, Grade 2 or Grade 3 CD was not seen at all in Non-CE patients. In general, the number of immune cells per area is considered to be correlated with the degree of histological inflammation. Thus, our results suggested CD with moderate or higher inflammation was observed only in CE patients.

The association between CE and habitual abortion has been reported [8, 10]. Considering these clinical data and the fact that there is a higher incidence of CD when CE patients miscarry, CD appears to be related to miscarriage.

Plasma cells, which are the basis of the diagnosis of CD, produce the antibody for some kind of antigen. In patients diagnosed with CD in the present study, maternal immunity may have reacted to the chorionic tissue (placental tissue) as an antigen during the course of miscarriage. Grade 1 CD was found in 4/13 Non-CE patients who miscarried. Such a histologically mild degree of CD may have been due to miscarriage.

In the total cases of Grade 2 and Grade 3 CD, the incidence of CE before the pregnancy was 100%. Thus, when Grade 2 or Grade 3 CD is found in the miscarriage tissue, it means that CE existed before pregnancy in all cases, although the number of samples was low in the present study. Specimens from miscarriage cases have been used to confirm the presence of chorionic tissue and the exclusion of chorionic diseases such as molar pregnancy. If specimens obtained from a patient who miscarried are examined for the presence of CD, this may provide a clue regarding the presence of CE before the pregnancy, which may be useful for the subsequent fertility treatment.

To the best of our knowledge, this is the first study in the world to study the relationship between CE and CD in miscarriage specimens in order to investigate the direct effects of CE on pregnancy. Currently, when diagnosed as CE, the patient is usually treated with antibiotics and then undergoes embryo transfer. When the present study was conducted, it was already beginning to be thought that antibiotics might be effective to improve clinical outcomes. The patients in the CE group extracted for the present study were those who did not want antibiotic treatment. In this sense, the cases who became pregnant but miscarried following their diagnosis with or without CE are few and extremely valuable. Furthermore, the miscarriage specimens of these patients were even more valuable, and the results of the analysis of the miscarriage specimens could be compared with the presence or absence of CE. This is the strength of the present study.

On the other hand, although the numbers of cases and controls satisfied the power analysis, they were relatively small. Patients who sought antibiotic treatment were treated with them and excluded from the study. Though this attitude is ethically correct, this means that the CE group did not reflect all CE patients in the study period. These are the limitations of the present study.

In the future, it is important to investigate the effects of antibiotic treatment for CE on the incidence of CD in this area of research. In addition, conversely, it is very important to study whether a patient diagnosed with CD in a miscarriage will subsequently be diagnosed with CE.

We hope that this research provides new insights into the relationships among CE, CD, and miscarriage.

## Conclusions

Histopathological analysis of specimens from cases of miscarriage showed clusters of plasma cells or five or more plasma cells found in the decidua in more than half of CE patients, whereas they were not found in Non-CE patients. This suggests that the effect of CE remains in the decidua during pregnancy. In addition, when the presence of a plasma cell cluster or five or more of plasma cells in 10 HPFs is confirmed histologically, the presence of CE before the pregnancy should be suspected. The analysis of the presence of CD in the specimens of miscarriage may help subsequent fertility treatment.

## Data Availability

We can provide the raw data. The datasets used and/or analyzed during the current study available from the corresponding author on reasonable request.

## Abbreviations

CD: Chronic deciduitis
CE: Chronic endometritis
HPF: high-power fields
NK cell: Natural killer cell
